# MSDmotif: exploring protein sites and motifs

**DOI:** 10.1186/1471-2105-9-312

**Published:** 2008-07-17

**Authors:** Adel Golovin, Kim Henrick

**Affiliations:** 1 EMBL Outstation, The European Bioinformatics Institute, Welcome Trust Genome Campus, Hinxton, Cambridge, UK

## Abstract

**Background:**

Protein structures have conserved features – motifs, which have a sufficient influence on the protein function. These motifs can be found in sequence as well as in 3D space. Understanding of these fragments is essential for 3D structure prediction, modelling and drug-design. The Protein Data Bank (PDB) is the source of this information however present search tools have limited 3D options to integrate protein sequence with its 3D structure.

**Results:**

We describe here a web application for querying the PDB for ligands, binding sites, small 3D structural and sequence motifs and the underlying database. Novel algorithms for chemical fragments, 3D motifs, ϕ/ψ sequences, super-secondary structure motifs and for small 3D structural motif associations searches are incorporated. The interface provides functionality for visualization, search criteria creation, sequence and 3D multiple alignment options. MSDmotif is an integrated system where a results page is also a search form. A set of motif statistics is available for analysis. This set includes molecule and motif binding statistics, distribution of motif sequences, occurrence of an amino-acid within a motif, correlation of amino-acids side-chain charges within a motif and Ramachandran plots for each residue. The binding statistics are presented in association with properties that include a ligand fragment library. Access is also provided through the distributed Annotation System (DAS) protocol. An additional entry point facilitates XML requests with XML responses.

**Conclusion:**

MSDmotif is unique by combining chemical, sequence and 3D data in a single search engine with a range of search and visualisation options. It provides multiple views of data found in the PDB archive for exploring protein structures.

## Background

Small sequence or structure protein fragments with highly conserved properties that may have important biological functions and have been used in tertiary structure and secondary structure prediction processes [1,2]. Although the application of structure motifs to a sequence where the structure is unknown requires additional information such as a global energy function, structure motifs in combination with sequence motifs can be mapped onto structures [3,4]. In addition, sequence and structure motifs have an application in drug design [5] when motifs map to active-sites and ligand binding sites. We have created an integrated resource of information about motifs and their environment from all Protein Databank (PDB) [6] entries. MSDmotif is organised using a number of categories to distinguish three general types of motifs: sequence motifs, small 3D structural motifs and super-secondary structure motifs.

Sequence motifs can be defined as a pattern without or with probabilistic preferences; for the latter use of hidden Markov models (HMM) [7] is often made. Sequence motif identification tools such as BLAST [8], FASTA [9], CLUSTALW [10] are available together with new emerging methods such as MEME [11]. In the MSDmotif database sequence motifs from the PROSITE [12] database have been incorporated.

Small 3D structural motifs consisting of up to 8 residues such as the beta-turn, are common in protein structures where they cover approximately 50% of the residues. These motifs can play a role in determining the conformation and specificity of enzyme active sites [13] and enzyme binding sites [14,15]. In addition they can have a role in protein folding and protein stability [16,17]. Small 3D structure motifs are classified through properties of hydrogen bonding, ϕ/ψ and χ angles independent of the sequence. These motifs have been classified into 13 distinct motifs: alpha-beta-motif, asx-motif, asx-turn, beta-bulge, beta-bulge-loop, beta-turn, catmat, gamma-turn, nest, schellmann-loop, st-motif, st-staple, st-turn [14,15,18-22] (see Additional file 1). We have mapped these motifs onto all PDB entries. MSDmotif contains a summary for each motif and provides a tool for the interactive analysis of their properties along with the ability for new motif discovery.

A previous study of super-secondary structure patterns[23] detailed eleven common such motifs through partial replacement of loops with a residue conformation identifier. These included the helix hair-pins [24] and beta hair-pins [25]. We have extended this method with a search method for secondary structure sequences by using the PROSITE format with additional restrictions on loop lengths between secondary structure moieties together with the ability to specify hydrogen and disulphide bonds.

MSDmotif contains integrated details about sequence, structure, the relative position and the neighbour environment of many motif types. The data are derived from the PDB and stored in a relational database, accessible through an interactive service. Search criteria can combine sequence motifs, structure motifs, protein sequence, 3D properties (like ϕ/ψ and χ angles, Cα and side-chain positions), secondary structure elements, 3D associations between motifs, protein side-chain and main-chain bonds and protein-ligand interactions. We also provide multiple sequence and multiple structure alignment tools.

## Implementation

### Phi-Psi ϕ/ψ search

The PDB can be queried using Phi-Psi angles (ϕ/ψ) fragments where the sequences of ϕ/ψ angles are a sequential representation of protein geometry and are directly comparable to coordinates. This type of search uses a sequential geometrical descriptor that results in linear dependency of the search task complexity from the number of elements. The challenge here is to look for similarity rather than an exact match through selecting the most likely allowed deviations and flexibility in length of the search fragment. The approach used is a refinement to our previously published method [26] for pattern searches based on an optimised database design and a web-application query generator to produce optimal SQL queries. The database consists of two tables, the first is indexed with each row representing a single amino-acid in 3D. This table has bi-tree indexes by unique residue identifier. The second contains sequential triples of amino-acids and is bitmap indexed on ϕ/ψ columns for each of the three residues. The query consists of multiple self joins of these tables.

### Sequence search

Sequence searching may be carried out on the complete PDB chains or more specifically on just the loop sequences where a loop is a non-helical, non β-strand fragment. Complex searches combining sequence and geometrical criteria are possible. We have used an implementation of PSI BLAST [27] integrated with the Oracle database engine, by parsing XML output from BLAST and streaming it into an Oracle transaction table.

### Super-secondary structure patterns search

Super-secondary structure patterns with associated geometrical characteristics queries are possible. The sequence pattern can be input using the PROSITE pattern notation where "or" can be specified as: [HE]LEL [HE], i.e. first element can be Helix or Strand, the second element is Loop, the third element is Strand, the fourth element is Loop and the fifth element is Helix or Strand. This simple pattern use may be combined with other constraints to build complex search criteria. For example an overlap of a secondary structure pattern with a protein sequence fragment can be constructed. The method is the same as we use for protein sequence pattern search described in [26].

### Small 3D motif associations search

Searching for associated 3D motifs is based on a relational database approach. We encode the search criteria in a separate dictionary table and then create a table for storing distances between all motifs within a protein chain. For ~50,000 PDB entries and with ~50% of the all residues involved in a motif gives a distance table of 300 million rows. The number of rows in the dictionary table is only ~30,000. The cardinality in this case is about 0.01%. For fast data access by a column with a low cardinality we create a bitmap index on this column and order the records in the distances table in accord with the indexed values. The SQL query is generated through path analysis on the graph of the motifs association where the target is to pick a path with the lowest cost. A similarity scoring system was introduced to order hits. A geometrical characteristic for each motif was defined using an alternative to RMSD [28]. We calculate (i) a vector from the first Cα to the last Cα for each motif, and (ii) the geometrical centre of all C,Cα,N atoms within the motif. Scoring is then calculated as the sum of the deviation of the geometric centre distances: abs(1-distance/original distance), and the cosine of the angle between each motif vector. The score is normalised to the total number of paired motifs. This scoring system counts only common features and has a good projection in 3D regardless of motif length, and is readily specified in SQL.

### 3D motifs regardless sequence

This search is based on Cα coordinates or end of side-chain coordinates. The end of side chain calculation is based on a 2D graph of an amino-acid to find the most remote atoms of the side-chain from the Cα, then the 3D coordinates of these atoms are averaged. The search is limited to a radius of 16 Angstrom. Scoring is calculated from the deviation of the base coordinates, and a residue direction vector going from Cα to the end of side-chain.

### Interactions

The PDB contains about 8000 unique small molecules [29], and we use this information to derive ligand, water, nucleic-acid and protein interactions based on the previously reported algorithm [26]. For better performance protein-protein interactions are separated into several database tables on the basis of main-chain/side-chain interactions, interactions within a chain and interactions between chains. We distinguish the following bond types:

• Covalent bonds (include disulphide bonds)

• Ionic bonds

• Hydrogen bonds (include salt bridges)

• van-der-Waals bonds

• Plane-plane (π electron) interactions

• Plane-atom interactions

• Unidentified interactions within 4.25 Angstroms.

Plane-plane interactions occur between chemical planar structures and between rings, similarly plane-atom interactions involve the above groups and an atom. In plane-plane interactions the preference is given to those where the planes or rings are parallel whether the second interaction with an atom is stronger when the atom approaches the plane orthogonally.

### Query generator

Creation of an optimal query is a crucial task for a complex system like MSDmotif where many different sub-queries can be combined into a search. The cost of each sub-query can be relatively high, for instance, protein sequence scanning using the N-glycosylation pattern – n{p} [st]{p}, has a high cost and gives many hits. Use of a standard SQL query would take an unacceptable time. Efficient queries require incorporation of Oracle instructions covering the execution plan and query assignment to the best index table. Such a complex query cannot rely on the Oracle cost optimiser and the database engine needs to be guided by Oracle optimiser hints. A popular solution for this problem is parameterisation of the query and tuning it manually to reach an acceptable performance. Assignment of optimiser hints is difficult. Design of an efficient general query system is challenging when combining chemical search, 3D motifs and sequences into a single query where the approach with pre-tuned queries leads to an exponential number of these queries. To achieve fast queries we developed a JAVA package that generates SQL with the necessary structure and optimiser hints leading to an optimal execution plan for the Oracle RDBMS. The approach wraps each search element into a sub-query, then the query generator applies a set of rules and uses preloaded statistics about the cost of a sub-query. First of all it selects a leading sub-query on the basis of the minimal number of expected rows. Then it decides which sub-queries must be executed independently. These second sub-queries will be combined using Cartesian or hash join where the latter is preferable. The rest of the sub-queries are assigned as dependant and they will be merged into the query using index access inside nested loops. A path analysis is carried out on the query graph making use of pre-determined table weighting and index weighting.

### Database and retrieval system

The database is derived from the PDB archive as a component of the Macromolecular Structure Database (MSD) [30] and is updated weekly with new entries. The design aim was to have a table structure optimised to serve queries. Therefore all features of motif definitions were reflected in the database scheme. For fast access motif tables are preloaded. The database was designed to cope with multiple table self-joins by the use of table normalisation and of duplicate tables storing both the data and the index. Textual information is stored in separate dictionary tables which are used for the on the fly hits annotation. The core PDB data is organised into four trees: proteins, nucleic-acids, bound-molecules and solvents. These trees span from chain to atomic levels, they are cross referenced by interaction tables on each level.

In the retrieval system operations such as sorting and grouping on the Oracle server can alter the execution plan and make it ineffective. To overcome such a challenge the client web-application orders, groups and normalises the hits, it uses numeric codes for retrieval and decodes these through cached dictionaries. The normalisation can be carried out on the basis of either CATH [31] or SCOP [32] or PFAM [33] families or by EC number [34], or by sequence identity. The importance of the normalisation flows from vast groups of closely related coordinate entries like NMR models, x-ray experiments with lisozimes, hemoglobinds and myoglobinds.

The hit list has a number of options to download sets of PDB structures and those fragments while individual structures can be downloaded from the corresponding links. Detail pages represent a number of views on the particular PDB entry with respect of the protein sequences, motifs, ligands and interactions. They provide facilities to download the structure in XML and PDB formats.

### Sequence alignment

The hit-list provides pseudo multiple sequence alignment. The sequences in the hit-list are aligned to the target sequence using PSI-BLAST pair-wise alignment. A variety of output formats for further visualisation of sequences alignment are given. Provisions for multiple visualization in Jalview [35] and Blixem [36] are available. Sequences may be aligned by either presenting the complete sequence aligned by the hit fragments, or by presenting alignment with the searched fragment only.

**Figure 1:**
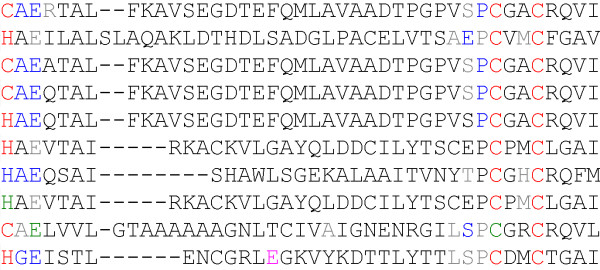
**Patterns multiple alignment**. Extract of a results page using the Cytidine and deoxycytidylate deaminases zinc-binding region signature, showing a pattern multiple alignment. The residue colour corresponds to a protein-ligand interaction.

Another feature is sequence alignment based on a search pattern target. Patterns can be flexible, e.g. the Cytidine and deoxycytidylate deaminases zinc-binding region signature, [CH]-[AGV]-E-x(2)-[LIVMFGAT]-[LIVM]-x(17,33)-P-C-x(2,8)-C-x(3)-[LIVM], has two flexible regions x(17,33) and x(2,8). We align matched fragments leaving gaps corresponding to these flexible regions. An example of alignment for this pattern is given in the Figure 1.

### 3D alignment

3D alignment is carried out by aligning the search elements. When the search includes an amino acid sequence then it is made of the BLAST alignment while when a motif is used then a residue correspondence is explicit in the hit list and an iterative alignment matrix is calculated until a minimal RMSD is reached.

### DAS server

The MSDmotif service includes DAS component that allows the facilities to be used by clients other than an internet browser. DAS, the Distributed Annotation System, is a simple client-server network protocol for exchange biological data [48,49,51].

Through the DAS registration server  MSDmotif provides DAS access to small 3D structure motifs. We have previously mapped the PDB protein chains to the corresponding UniProt [37] entries and MSDmotif uses these mapping to provide a dual access DAS server. In DAS terms the MSDmotif DAS server supports two coordinate systems: PDB structure and UniProt sequence, such that our data can be presented in the DAS clients DASTY [50], ENSEMBL [38] and SPICE [39].

## Results and discussion

**Figure 2:**
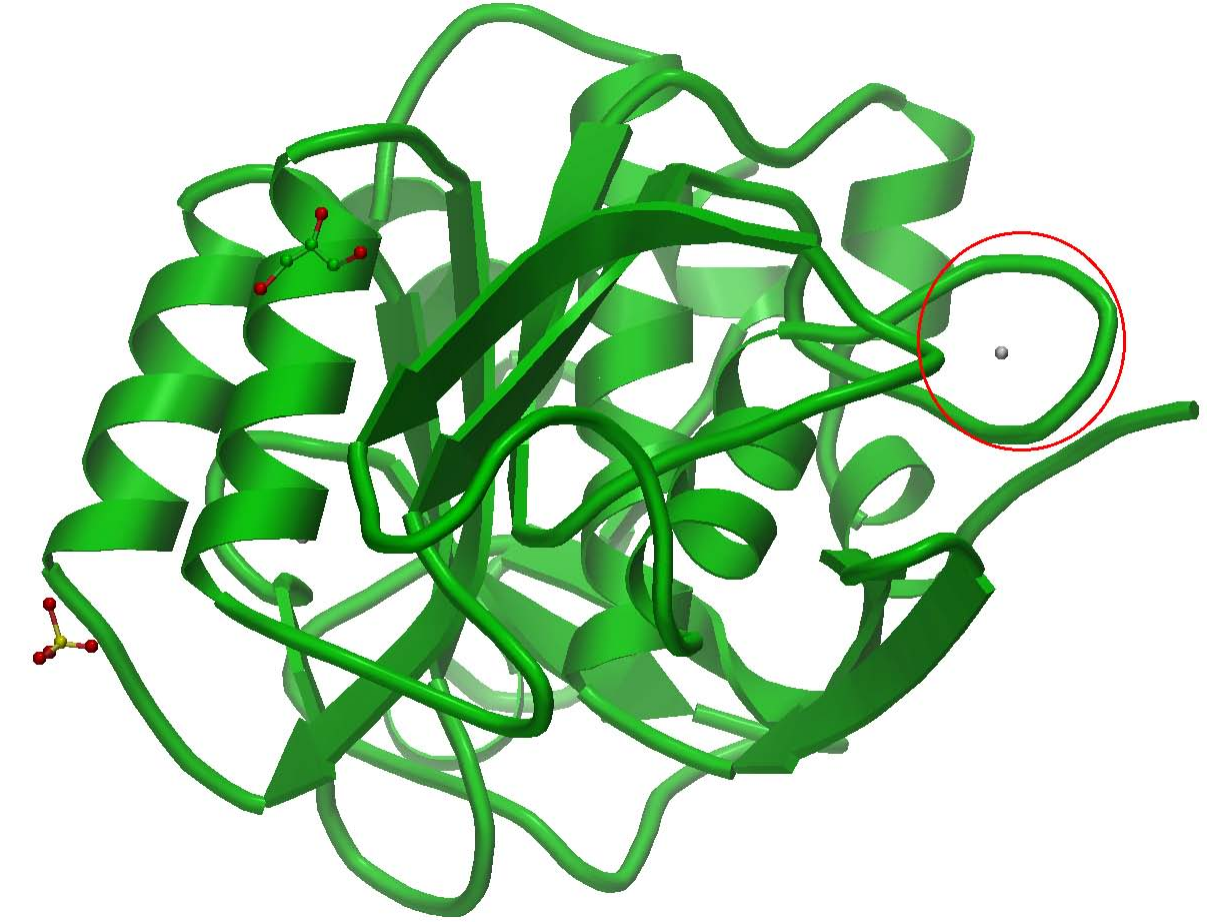
**1gci PDB entry**. 1gci PDB entry, a member of Subtilases SCOP family with the Calcium binding loop in the red circle. The picture was taken from EBI-AstexViewer TM+ [45,46].

**Figure 3:**
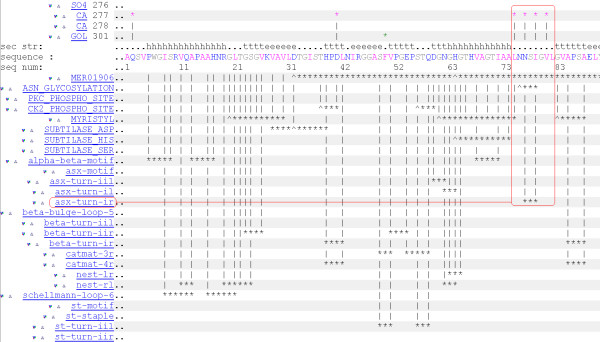
**MSDmotif PDB detail page**. shows part of the MSDmotif PDB entry page for 1gci giving the sequence annotation with the Calcium binding loop highlighted in red as residues 75–82 (LNNSIGVL) of chain A. The Calcium ion binds residues 75,77,79,81 which contain an Asx-turn (residues 77–79). Vertical bars (|) represent the start and end of a particular motif while the asterisk's (*) represent the extent of the motif and underline the sequence. .

**Figure 4:**
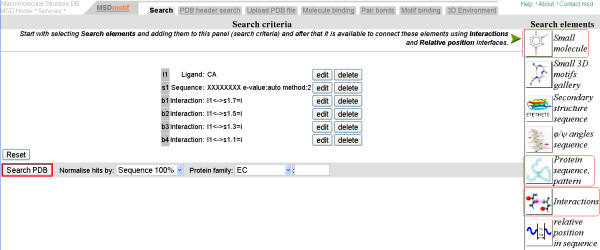
**Search interface**. Search interface with calcium binding site search criteria where the ligand is Calcium, the sequence pattern is xxxxxxxx and residues 1,3,5,7 of the pattern coordinate the ion. In the right column, the highlighted interfaces were used to form the query.

**Figure 5:**
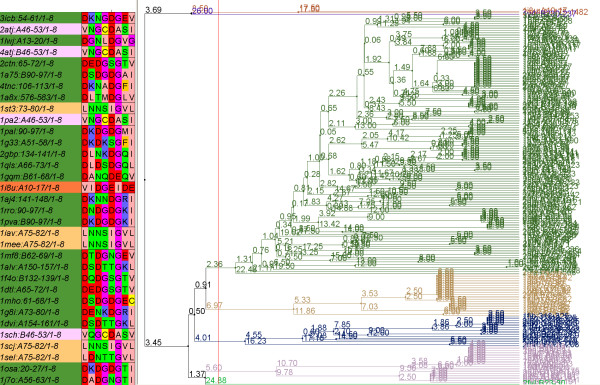
**Clustering sequences from the search result**. Jalview presentation of the search results. To the right there is a fragment of 8 residues long sequences and to the left there is the clusters hierarchical tree. The red line trims the tree into two brunches where the top one consists of sequences starting with A, I, L, V and the largest one of sequences starting with D,N.

We discuss how to apply MSDmotif tools for sequence and 3D structural motifs determination through an example. Consider the Calcium-binding loop found in PDB entry 1gci [42], a member of the Subtilases SCOP family. It is shown in the Figure 2. Calcium binding has been the subject of a number of studies [40,41]. MSDmotif provides an extensive analysis of a binding site and its environment, together with annotation of protein ligand interactions, PROSITE patterns, MEROPS [43] sites, Catalytic sites [44], and motifs as shown in Figure 3 for PDB entry 1gci. An example of using MSDmotif is to take the 1gci calcium binding residues, LNNSIGVL, and represent this simply as the non-specific eight residues "xxxxxxxx", but keep the condition that the residues 1,3,5,7 bind the ion. A query can be built using the sequence, the small molecule and the interaction interfaces from the search tab as shown on the Figure 4. Submitting this query gives aligned sequence fragments in CLUSTALW format. The alignment can be viewed with Jalview and clustered using the average distance determined by the BLOSUM62 matrix. As shows the Figure 5 there is a division of the clusters into major groups with one starting with hydrophilic amino-acids (D-Aspatic or N-Asparagine) and the others starting with hydrophobic amino-acids (A-Alanine, L-Leucine, I-Isoleucine and V-Valine).

Iterating the "xxxxxxxx" pattern with [DN]xxxxxxx or with [AILV]xxxxxxx results in the following patterns:

• [DN]{GYW}[DQNTS]G[DQNTS]G{GPW}[ACGHILV]

• [AILV][DILNQS][DGNPV][ACDGST][DEGITV][GARW][EDFSV][EILNVW]

**Figure 6:**
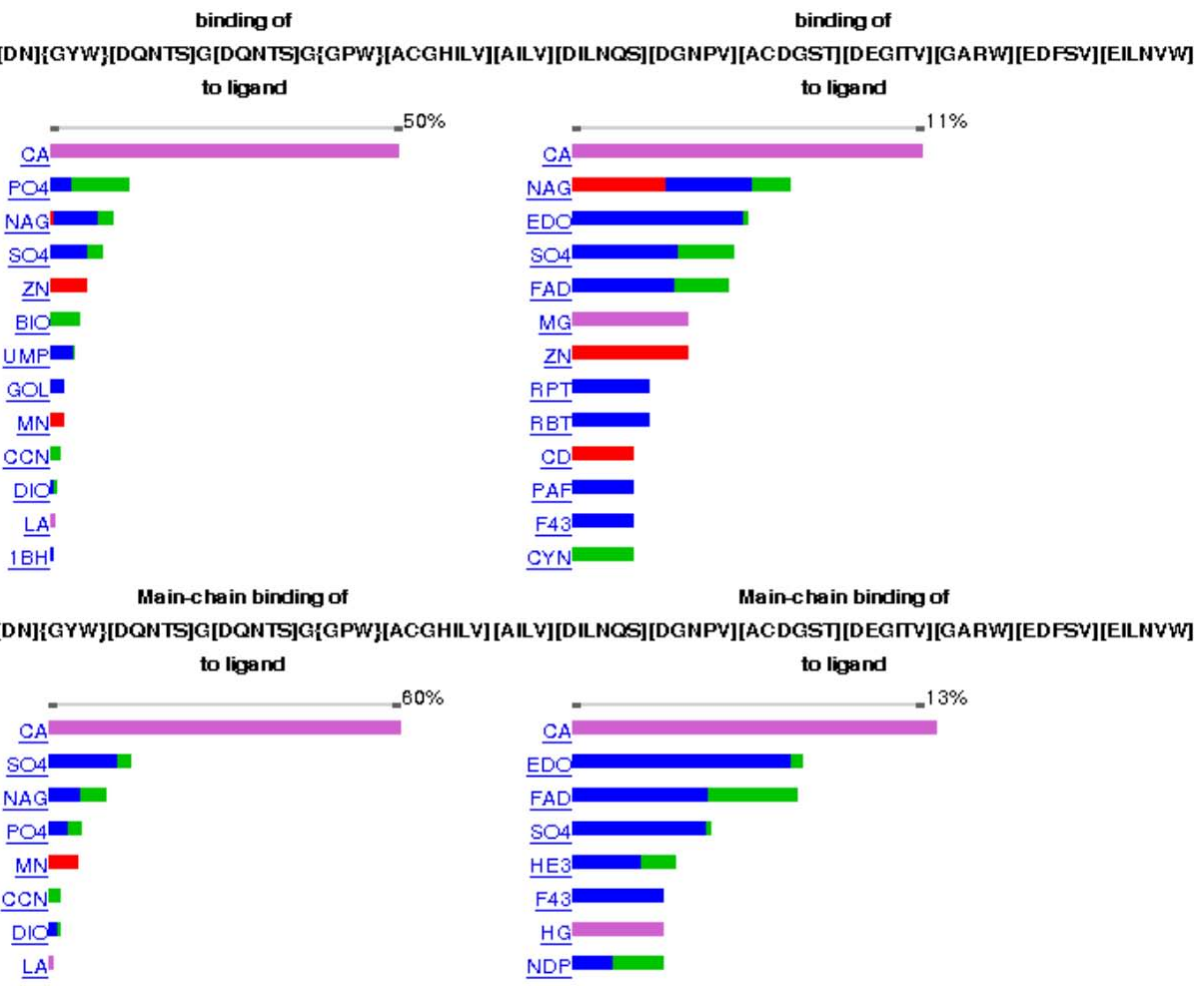
**Ligand binding sensitivity of the new patterns**. Ligands are referred by those three letters code. The charts are obtained by the use of "Motif binding statistics" interface. The colour of the bars corresponds to the bond types where red used for covalent bonds, pink for ionic bonds, blue for hydrogen bonds and green for van-dre-Waals bonds. The patterns become more selective when the interactions are restricted to main-chain only. . .

The starting sequence contained an Asx-turn and although the subsequent search did not use this information the derived patterns contain either an Asx-turn or its twin the ST-turn. The first pattern: [DN]{GYW}[DQNTS]G[DQNTS]G{GPW}[ACGHILV] has an Asx-turn at positions 1, 3 and 5. This pattern is similar to PROSITE PS00018 [47] pattern for the EF-hand calcium-binding domain. The second pattern: [AILV][DILNQS][DGNPV][ACDGST][DEGITV][GARW][EDFSV][EILNVW] has an ST-turn at positions 2,3 and 5. Both patterns can be analysed for ligand binding preferences as shown in Figure 6.

**Figure 7:**
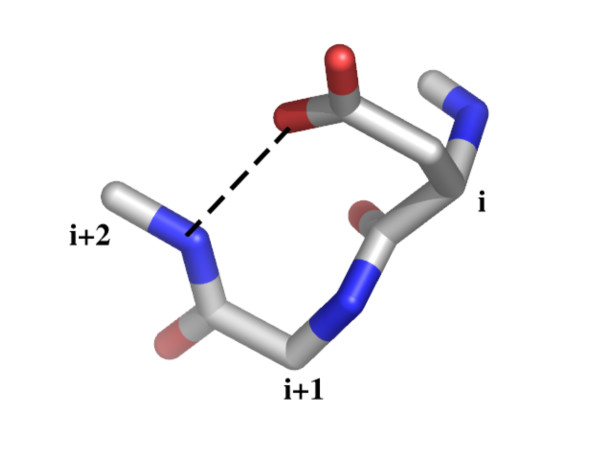
**Asx-turn**. 3D structural motif: Asx-turn. Residue(i) is Aspartate or Asparagine and the side-chain O of residue(i) is H-bonded to the main-chain NH of residue (i+2). There are restrictions on the φ,ϕ,χ angles. The definition and statistics can be found at: .

**Figure 8:**
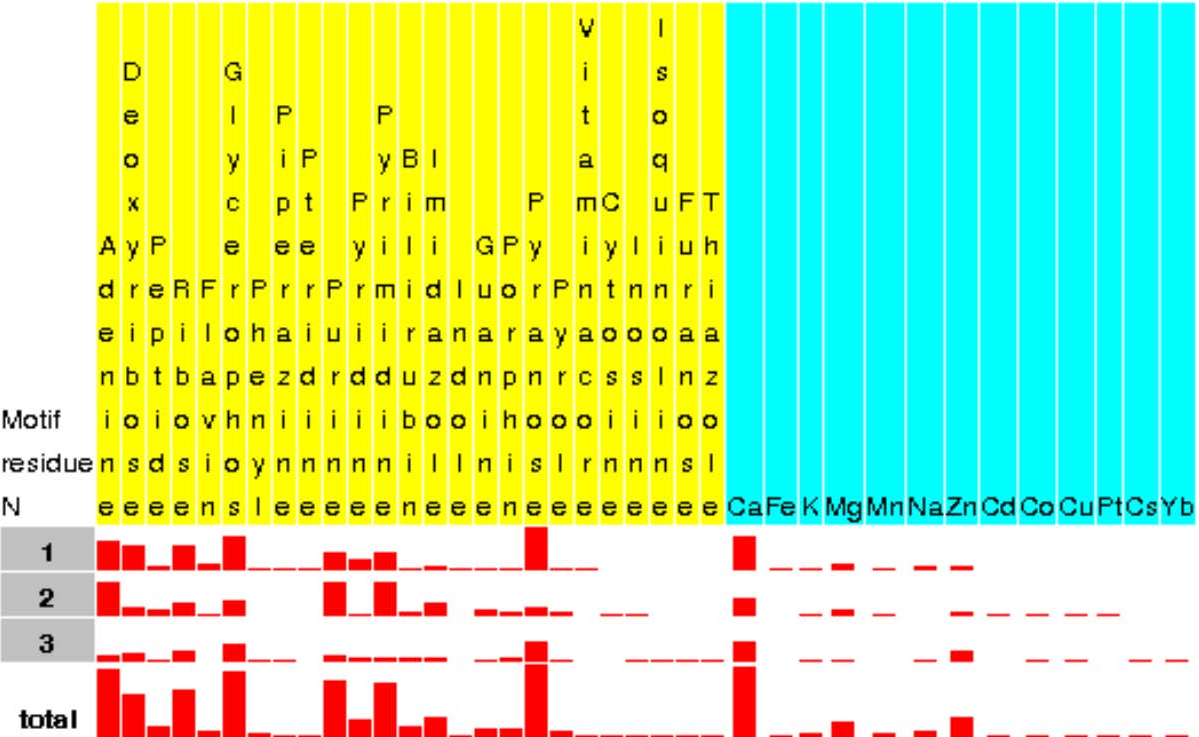
**Asx-turn ligand fragments binding**. Asx-turn ligand fragments binding as given by the statistics and 3D structural motif definition page .

**Figure 9:**
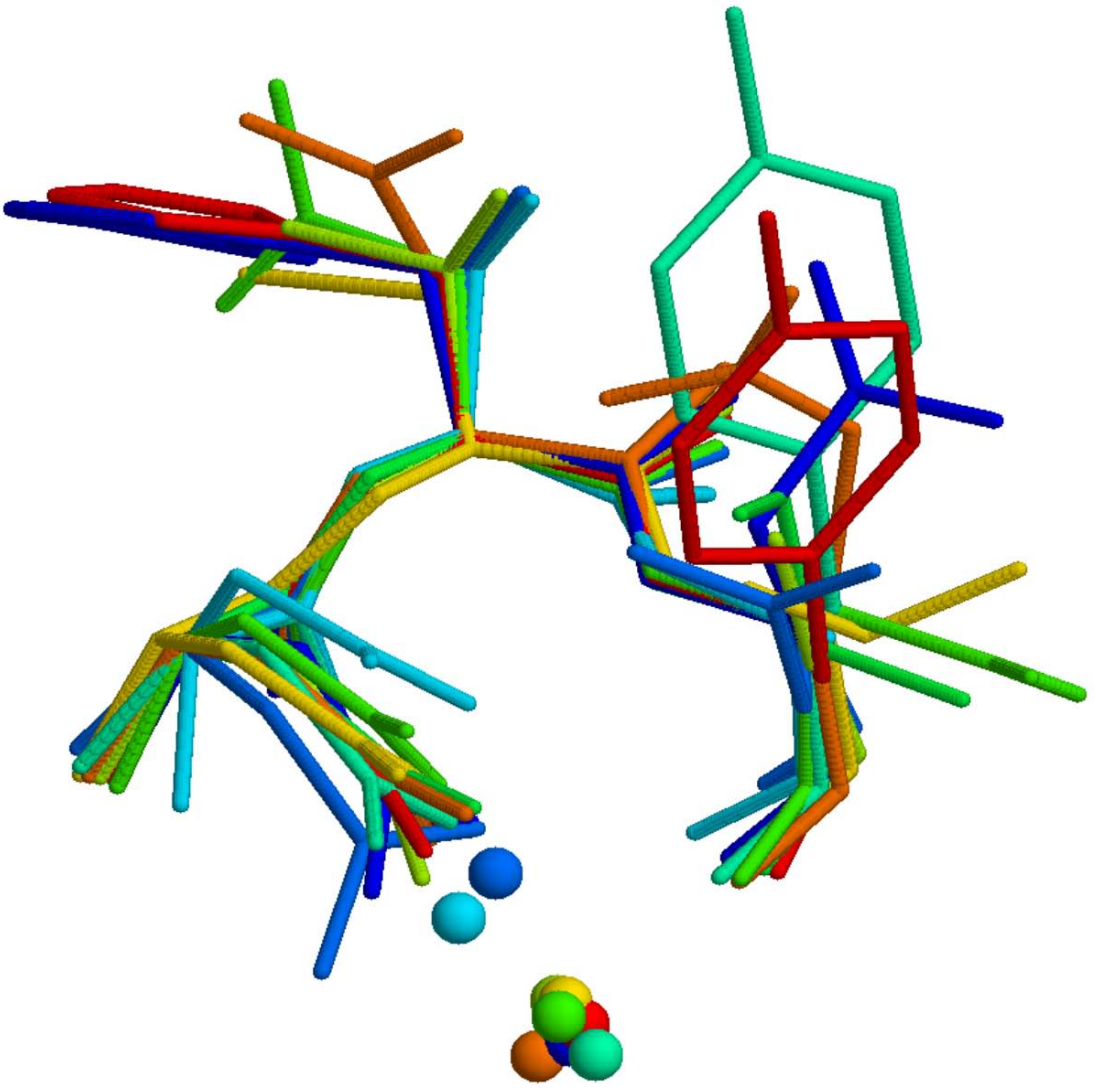
**3D multiple alignment of Asx-turns**. 3D multiple alignment of Asx-turns associated with a Calcium ion where the majority of the ions are coordinated within the turn.

The derived patterns show a high sensitivity and specificity to bind Calcium and other ions and can be further queried as regards their 3D conformations. The Calcium binding loop in PDB 1gci is associated with Asx-turn, residues 77–79. The motif is shown in the Figure 7 and is highlighted in a sample structure in the Figure 3. It is possible to start searching with the Asx-turn. This is a common motif found in about 2200 (70%) SCOP families. The definition and statistics for asx-turn motifs found in the PDB archive can be viewed by selecting the corresponding link on the PDB entry sequence detail page in Figure 3. The statistics include ligand binding sensitivity to chemical fragments as presented in the Figure 8. It shows the Calcium ion as a frequently observed ligand interacting with residues 1 and 3. The asx-turn motif appears to have a high binding sensitivity to Calcium ions with the interaction occurring mostly between residue 1 and 3. The interaction chart shown in Figure 8 can be used as a query interface to give a new hit-list. The subsequent matches can be aligned in 3D as shown in Figure 9 giving the distribution of Calcium ions about the motif.

**Figure 10:**
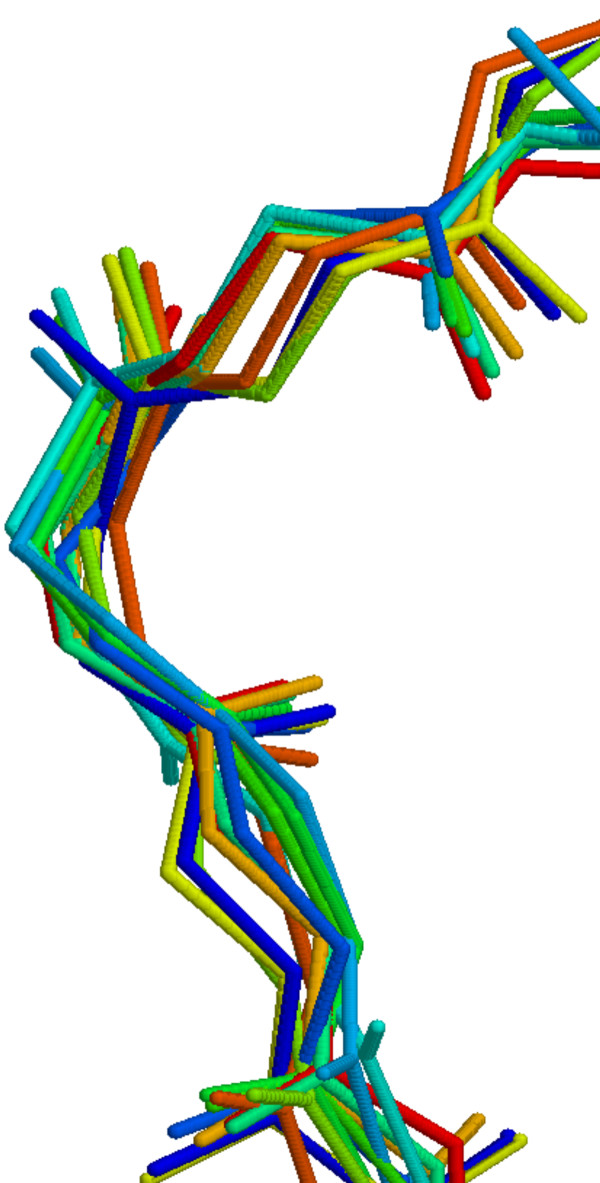
**3D alignment of hits found by ϕ/ψ search**. 3D alignment of hits found by ϕ/ψ search using dihedral angles of a sample Asx-turn from 1gci PDB entry.

Further queries made by correlating SCOP family data, Asx-turn presence and Calcium ions, show that 40% of the SCOP families, have this motif interacting with the ion. Interestingly extending the query by removing constraints that the first residue must be Aspatate acid or Asparagine acid and applying instead restrictions on the ϕ/ψ angles for all three residues using the ϕ/ψ search option. This approach takes the starting ϕ/ψ values from the resulting web page (Figure 3) for residues 77 and 79 with the constraints that the angles deviate by ± 60 degrees and we limit matches to be from different SCOP families only. This gives ~2350 (76%) SCOP families and shows a good 3D alignment [see Figure 10]. The figure shows main-chain only of top 20 hits by PDB resolution. Here THR is the most common first residue suggesting a similarity between the ST-turn and the Asx-turn. However there is no overall sequence commonality and the first residue is variable indicating that the sequence specific ST-turn and Asx-turn's have a common 3D conformation that is non-specific for sequence.

### Summary of the MSDmotif features

#### Search elements

• Small molecules – draw a chemical fragment using JME or use a molecules code

• Sequence patterns – submit a PROSITE format pattern

• Sequences – submit a protein sequence to NCBI PSI-BLAST integrated search

• ϕ/ψ sequences – submit a sequence of dihedral angles with a given tolerance

• PROSITE motifs – submit a motif by PROSITE code

• MEROPS sequences – submit a preloaded MEROPS sequence by its code

• Catalytic sites – submit a preloaded Catalytic site by its code

• Small 3D structural motifs – choice from the gallery of 3D structural motifs

• Super-secondary structures – submit a sequence or a pattern made of secondary structure elements.

• Small 3D motif associations – submit a group of motifs where relative position in 3D is fixed.

• 3D motifs by Cα or end of side-chain coordinates – sequence unrelated 3D search by atom coordinates.

#### Constraints on search elements

• Interactions between ligands, proteins, nucleic-acids and solvents.

• Relative position of motifs in a protein sequence

#### Statistics search

• Molecule binding statistics where the distribution is done over:

○ PROSITE motifs

○ Small 3D structural motifs

○ Secondary structure elements

○ Protein amino-acids, Nucleic-acids and water molecules. This statistics are available on residue and on atomic levels

○ Sets of environment amino-acids

• Sequence pattern, PROSITE, 3D structural motifs, secondary structure elements binding statistics with respect

○ Ligands

○ Modified amino-acids

○ Nucleic-acids

○ PROSITE motifs

○ Small 3D structural motifs

○ Secondary structure elements like helix, strand, loop

• Small 3D motif Ramachadran plots for each residue

• Small 3D motif sequence distributions

• Small 3D motifs parameter distributions and correlations

#### Supported desktop visualisation tools

• 3d structure visualisation – RasMol, Jmol, EBI-Astex viewer

• Multiple sequence alignment – JalView, Blixem

• Chemical fragments – Java Molecule Editor (JME)

## Conclusion

The service brings together many aspects of protein structures. It can be used by crystallographers to search whether interesting fragments of those structures have been crystallized and what were the experiment details. Scientists can use it to understand interconnection between protein 3D structure and the sequence. Multiple views on the data help to navigate in multi-dimensional space made of chemical 2D structures, protein sequences, tertiary and quaternary structures. Structural biology PHD students can complete thesis in shorter terms with a higher quality and scientific content.

## Availability and requirements

Project name: MSDmotif

Project home page: 

Operating systems: Platform independent

Programming languages: C++, Java, JSP, SQL, PL/SQL

Other requirements: Internet Browser IE 6.x or Mozilla 4.0, for in house installation:Tomcat 5.x, Oracle 9.x

License: GNU GPL

Database documentation: 

Case studies: 

## Authors' contributions

AG developed the service with the underlying database as well as wrote the draft of the manuscript.

KH contributed many ideas to the service and take major role in editing and rewriting the manuscript.

## Supplementary Material

Additional file 1 Appendix A. Small 3D structural motifs Click here for file
