# Supplementary material for: MSDmotif: exploring protein sites and motifs
Source: BMC Bioinformatics. 2008 Jul 17;9:312. doi: 10.1186/1471-2105-9-312 (PMC2491636; doi:10.1186/1471-2105-9-312)
Supplement: Additional file 1 — Appendix A. Small 3D structural motifs [file 1471-2105-9-312-S1.pdf]

Appendix A

Small 3D structural motifs

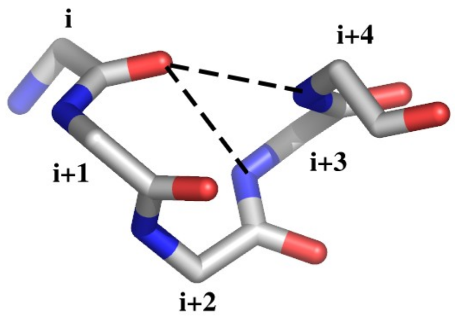

Alpha-Beta motif

A motif of five consecutive residues and two H-bonds in which:

- H-bond between CO of residue(i) and NH of residue(i+4)
- H-bond between CO of residue(i) and NH of residue(i+3)
- φ angles of residues(i+1), (i+2) and (i+3) are negative.

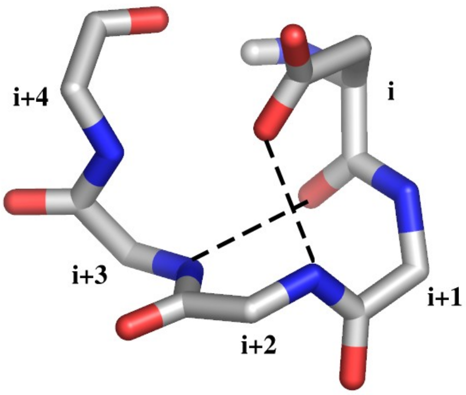

Asx-motif

A motif of five consecutive residues and two H-bonds in which:

- residue(i) is Aspartate or Asparagine (Asx)
- side-chain O of residue(i) is H-bonded to the main-chain NH of residue(i+2) or (i+3)
- main-chain CO of residue(i) is H-bonded to the main-chain NH of residue(i+3) or (i+4)

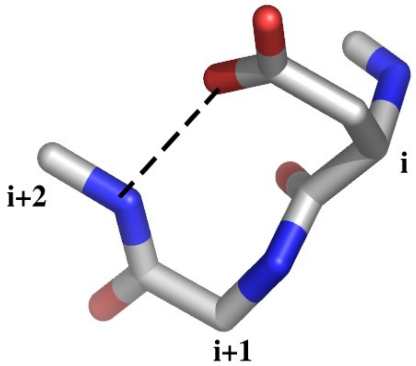

Asx-turn

A motif of three consecutive residues and one H-bond in which:
- residue(i) is Aspartate or Asparagine (Asx)
- the side-chain O of residue(i) is H-bonded to the main-chain NH of residue(i+2).

Sub-categories

```
Type I
   residue(i):   -140° < χ1 - 120° < -20°
                  -90° < ψ + 120° < 40°
   residue(i+1): -140° < φ < -20°
                  -90° < ψ < 40°
Type II
   residue(i):   -140° < χ1 - 120° < -20°
                   80° < ψ + 120° < 180°
   residue(i+1):   20° < φ < 140°
                  -40° < ψ < 90°
Types I' and II'
   Left-handed form of Type I and II consequently
```

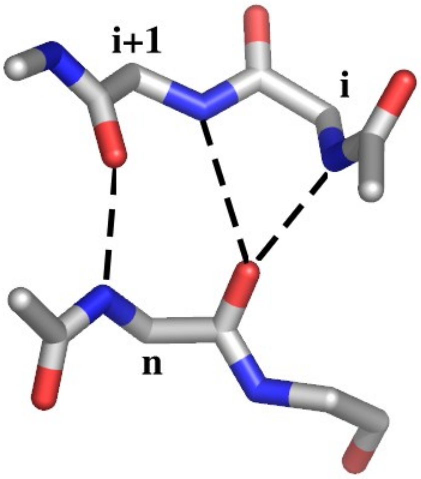

Beta-bulge

A motif of three residues within a β-sheet in which the main chains of two consecutive residues are H-bonded to that of the third, and in which the dihedral angles are as follows:

```
   residue(i):   -140° < φ < -20°     -90° < ψ < 40°
   residue(i+1): -180° < φ < -25° or  120° < φ < 180°
                   40° < ψ < 180° or -180° < ψ < -120°
```

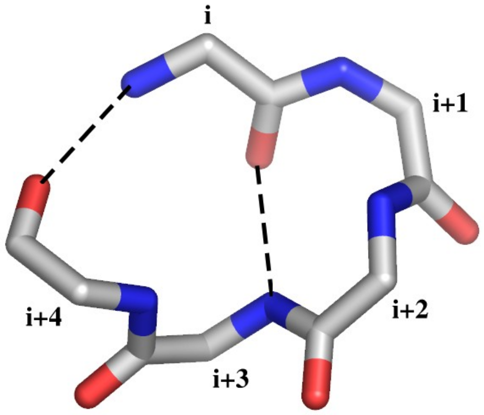

Beta-bulge loop

A motif of three residues within a β-sheet consisting of two H-bonds in which:

- the main-chain NH of residue(i) is H-bonded to the main-chain CO of residue(i+4) (Type 1) or residue(i+5) (Type 2)
- the main-chain CO of residue i is H-bonded to the main-chain NH of residue(i+3) (Type 1) or residue(i+4) (Type 2)

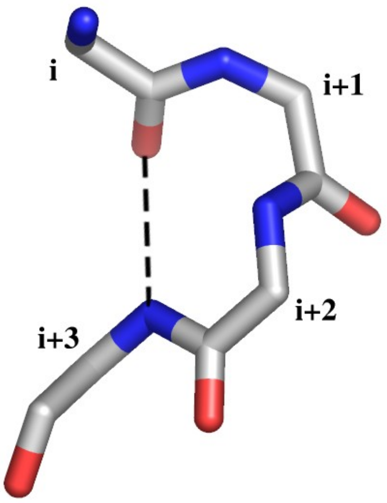

Beta-turn

A motif of four consecutive residues that may contain one H-bond, which, if present, is between the main-chain CO of the first residue and the main-chain NH of the fourth. It is characterized by the dihedral angles of the second and third residues, which are the basis for sub-categorization:

Sub-categories

```
Type I
   residue(i):   -140° < φ < -20°   -90° < ψ < 40°
   residue(i+1): -140° < φ < -20°   -90° < ψ < 40°
Type II
   residue(i):   -140° < φ < -20°    80° < ψ < 180°
   residue(i+1):   20° < φ < 140°   -40° < ψ < 90°
Type I' and II'
   Left-handed form of Type I and II consequently
```

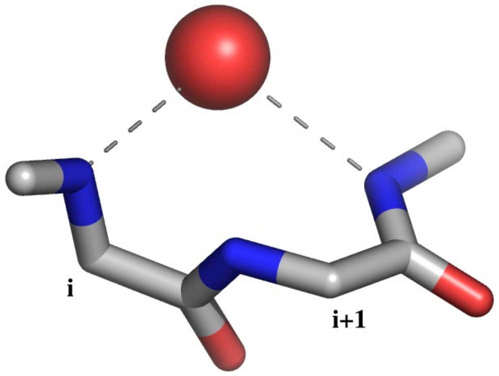

Nest

A motif of two consecutive residues with dihedral angles as follows (for the RL form):

Sub-categories

Type RL
   residue(i): $-140^\circ < \varphi < -20^\circ$ $-90^\circ < \psi < 40^\circ$
   residue(i+1): $20^\circ < \varphi < 140^\circ$ $-40^\circ < \psi < 90^\circ$
Type LR
   In LR nests the φ and ψ values for (i) and (i+1) are interchanged.

Nest should not have Proline as any residue.

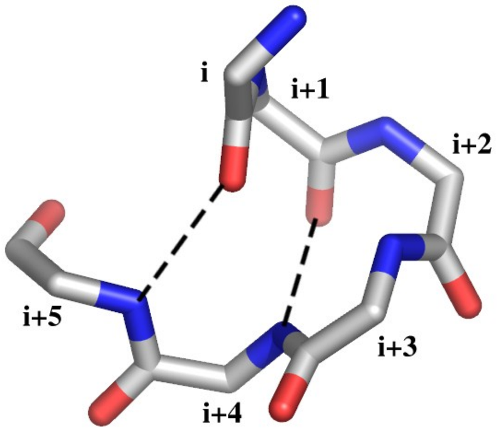

Schellmann loop

A motif of six consecutive residues (common type) or seven consecutive residues (wide type) that contains two H-bonds in which:

- the main-chain CO of residue(i) is H-bonded to the main-chain NH of residue(i+5) (common type) or residue(i+6) (wide type)
- the main-chain CO of residue(i+1) is H-bonded to the main-chain NH of residue(i+4) (common type) or residue(i+5) (wide type)

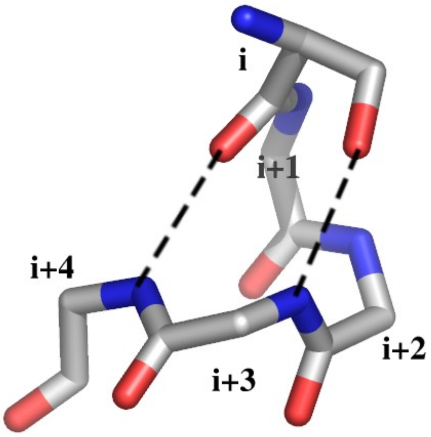

ST-motif

A motif of five consecutive residues and two H-bonds in which:

- residue(i) is Serine (S) or Threonine (T)
- side-chain O of residue(i) is H-bonded to the main-chain NH of residue(i+2) or (i+3)
- main-chain CO of residue(i) is H-bonded to the main-chain NH of residue(i+3) or (i+4)

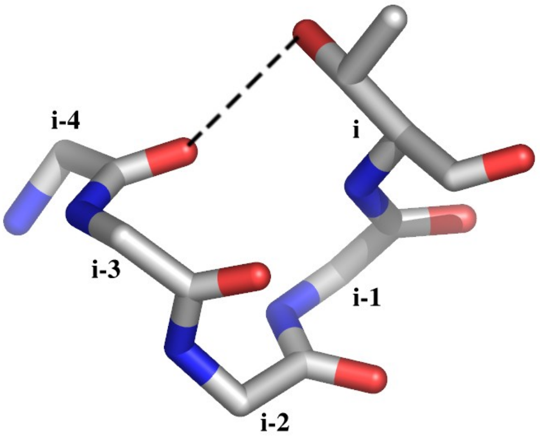

ST-staple

A motif of four or five consecutive residues and one H-bond in which:

- residue(i) is Serine (S) or Threonine (T)
- the side-chain OH of residue(i) is H-bonded to the main-chain CO of residue(i–3) or (i–4)
- φ angles of residues(i–1), (i–2) and (i–3) are negative.

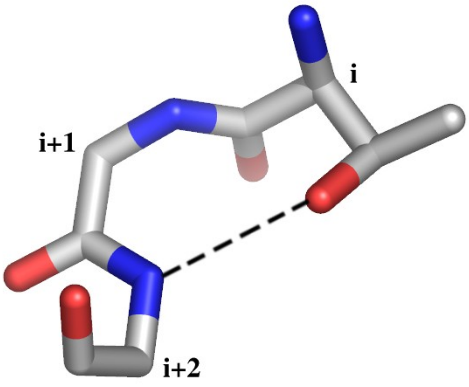

ST-turn

A motif of three consecutive residues and one H-bond in which:

- residue(i) is Serine (S) or Threonine (T)
- the side-chain O of residue(i) is H-bonded to the main-chain NH of residue(i+2).

Sub-categories

```
Type I
   residue(i):   -140° < χ1 - 120° < -20°
                  -90° < ψ + 120° < 40°
   residue(i+1): -140° < φ < -20°
                  -90° < ψ < 40°
Type II
   residue(i):   -140° < χ1 - 120° < -20°
                   80° < ψ + 120° < 180°
   residue(i+1):   20° < φ < 140°
                  -40° < ψ < 90°
Types I' and II'
   Left-handed form of Type I and II consequenly
```

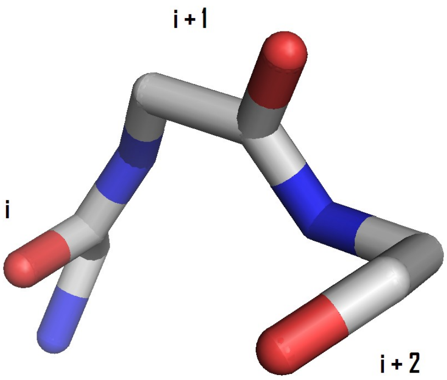

Catmat

A motif of 3 or 4 consecutive residues with dihedral angles as follows:

```
   residue (i-1): no limitations on φ/ψ
   residue   (i):  -120° < φ < -60°    -50° < ψ < 30°
   residue (i+1):  -100° < φ < -50°    110° < ψ < 170°
```

or

```
   residue (i-1): no limitations on φ/ψ
   residue   (i):  -120° < φ < -60°    -50° < ψ < 30°
   residue (i+1):  -120° < φ < -60°    -50° < ψ < 30°
   residue (i+2):  -100° < φ < -50°    110° < ψ < 170°
```

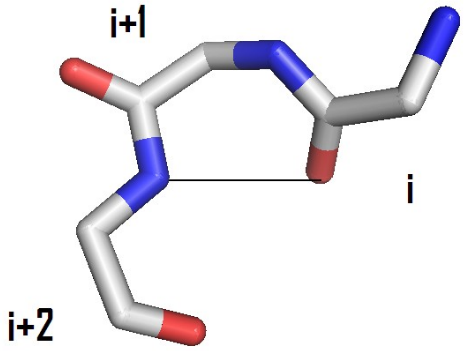

Gamma-turn

A motif of three consecutive residues i, i+1, i+2 and one H-bond in which:
- the main-chain O of residue(i) is H-bonded to the main-chain NH of residue(i+2).

Sub-categories

Type classic
residue(i+1): 35° < φ < 115° -104° < ψ < -24°
Type inverse
residue(i+1): -115° < φ < -35° 24° < ψ < 104°
